# Combination Chemotherapy with Suboptimal Doses of Benznidazole and Pentoxifylline Sustains Partial Reversion of Experimental Chagas' Heart Disease

**DOI:** 10.1128/AAC.02123-15

**Published:** 2016-06-20

**Authors:** Glaucia Vilar-Pereira, Isabela Resende Pereira, Leonardo Alexandre de Souza Ruivo, Otacilio Cruz Moreira, Andrea Alice da Silva, Constança Britto, Joseli Lannes-Vieira

**Affiliations:** a Laboratório de Biologia das Interações, Instituto Oswaldo Cruz/Fiocruz, Rio de Janeiro, RJ, Brazil; b Laboratório de Biologia Molecular e Doenças Endêmicas, Instituto Oswaldo Cruz/Fiocruz, Rio de Janeiro, RJ, Brazil; c Departamento de Patologia, Faculdade de Medicina, Universidade Federal Fluminense, Niterói, RJ, Brazil

## Abstract

Chronic chagasic cardiomyopathy (CCC) progresses with parasite persistence, fibrosis, and electrical alterations associated with an unbalanced immune response such as high plasma levels of tumor necrosis factor (TNF) and nitric oxide (NO). Presently, the available treatments only mitigate the symptoms of CCC. To improve CCC prognosis, we interfered with the parasite load and unbalanced immune response using the trypanocidal drug benznidazole (Bz) and the immunoregulator pentoxifylline (PTX). C57BL/6 mice chronically infected with the Colombian strain of Trypanosoma cruzi and with signs of CCC were treated for 30 days with a suboptimal dose of Bz (25 mg/kg of body weight), PTX (20 mg/kg), or their combination (Bz plus PTX) and analyzed for electrocardiographic, histopathological, and immunological changes. Bz (76%) and Bz-plus-PTX (79%) therapies decreased parasite loads. Although the three therapies reduced myocarditis and fibrosis and ameliorated electrical alterations, only Bz plus PTX restored normal heart rate-corrected QT (QTc) intervals. Bz-plus-PTX-treated mice presented complementary effects of Bz and PTX, which reduced TNF expression (37%) in heart tissue and restored normal TNF receptor 1 expression on CD8^+^ T cells, respectively. Bz (85%) and PTX (70%) therapies reduced the expression of inducible nitric oxide synthase (iNOS/NOS2) in heart tissue, but only Bz (58%) reduced NO levels in serum. These effects were more pronounced after Bz-plus-PTX therapy. Moreover, 30 to 50 days after treatment cessation, reductions of the prolonged QTc and QRS intervals were sustained in Bz-plus-PTX-treated mice. Our findings support the importance of interfering with the etiological agent and immunological abnormalities to improve CCC prognosis, opening an opportunity for a better quality of life for Chagas' disease (CD) patients.

## INTRODUCTION

Chagas' disease (CD) is a chronic neglected tropical disease caused by the protozoan parasite Trypanosoma cruzi. Chronic chagasic cardiomyopathy (CCC), the main cause of morbidity and mortality among CD patients, is characterized by low-grade inflammation, parasite persistence, and progressive fibrosis with remodeling of the myocardium and vasculature, which commonly causes heart failure and sudden death (1, 2). Given the lack of an effective specific therapy, CCC is treated similarly to other heart failure syndromes (1). Thus, a current challenge is to find an effective treatment for the millions of chronic CD patients.

The pathogenesis of CCC is thought to be related to parasite-dependent myocardial damage and immune-mediated myocardial injury (2–6). Increased levels of tumor necrosis factor (TNF) and nitric oxide (NO) in plasma are associated with the severity of heart dysfunction in CD patients (4–6). NO and TNF have been proposed to play a pivotal role in parasite growth control in acute T. cruzi infection (7). However, administration of the anti-TNF antibody infliximab to acutely infected mice did not aggravate the parasite burden but reduced fibrosis and cardiomyocyte damage (8). Furthermore, in the chronic phase of T. cruzi infection, the blockage of TNF repositioned immunological abnormalities and ameliorated CCC without interfering with parasite control (9). *In vitro*, TNF upregulates T. cruzi-triggered inducible NO synthase (iNOS/NOS2) expression and NO production by cardiomyocytes (10). NO is an important trypanocidal agent (7); however, persistently high NO levels were related to CCC severity in CD patients and nonhuman primate and mouse models of experimental Chagas' heart disease (6, 11–13). In addition, in rhesus monkeys, the severity of CCC was associated with the intensity of iNOS/NOS2-positive (iNOS/NOS2^+^) cell-containing myocarditis (12). Altogether, these findings broadcasted the idea that the modulation of TNF and NO inflammatory circuits may prevent CCC progression.

Although CD was identified more than a hundred years ago, the current therapeutic options for this condition are limited and sometimes poorly tolerable (1). Thus, combination therapy confronting the proposed basis for CD pathogenesis, the parasite and an unbalanced immune response, emerges as an alternative to improve treatment efficacy and reduce the dose, treatment period, and toxicity of benznidazole (Bz), the main drug available to treat CD (14, 15). Pentoxifylline (PTX), a methylxanthine phosphodiesterase inhibitor, is commonly used for the treatment of peripheral vascular disease and shows potential as an anti-inflammatory and cardioprotective agent (16, 17). In experimental CCC, PTX therapy reduced CD8^+^ T-cell abnormalities and TNF receptor 1 (TNFR1) overexpression and ameliorated heart function without modifying the parasite load (18). There is no solid scientific evidence about the use of benznidazole in the chronic phase of T. cruzi infection (14, 15). A recent report showed that Bz administration to patients with established CCC reduced the circulating parasite load but did not reduce cardiac clinical deterioration in a 5-year follow-up (19); however, the effect of Bz therapy in patients with incipient or mild chagasic cardiomyopathy remains to be explored. Bz treatment in the acute phase of infection prevented severe experimental CCC in mice (20) and dogs (21). Therefore, based on the hypothesis that CCC pathogenesis relies on T. cruzi persistence and parasite-driven deregulation of the immune response (2–6, 18, 22), we tested the idea that the trypanocidal drug Bz combined with the immunomodulatory agent PTX may improve CCC prognosis. For this, infected mice with signs of CCC were subjected to Bz, PTX, or Bz-plus-PTX therapeutic schemes and analyzed for parasite load, immunological biomarkers (TNF, TNFR1, iNOS/NOS2, and NO), and electrocardiographic abnormalities.

## MATERIALS AND METHODS

### Ethical information.

This study was carried out in strict accordance with recommendations in the *Guide for the Care and Use of Laboratory Animals* of the Brazilian National Council of Animal Experimentation (http://www.cobea.org.br/) and Federal Law 11.794 (8 October 2008). The Institutional Committee for Animal Ethics of Fiocruz (CEUA-Fiocruz L004/09 and LW10/14) approved all experimental procedures used in the present study. Data in each figure represent results from two or three independent experiments (P4, D2, D3G, and D4, Experiment Register Books 41, 49, 53, and 57; LBI/IOC-Fiocruz).

### Experimental design and T. cruzi infection.

**FIG 1:**
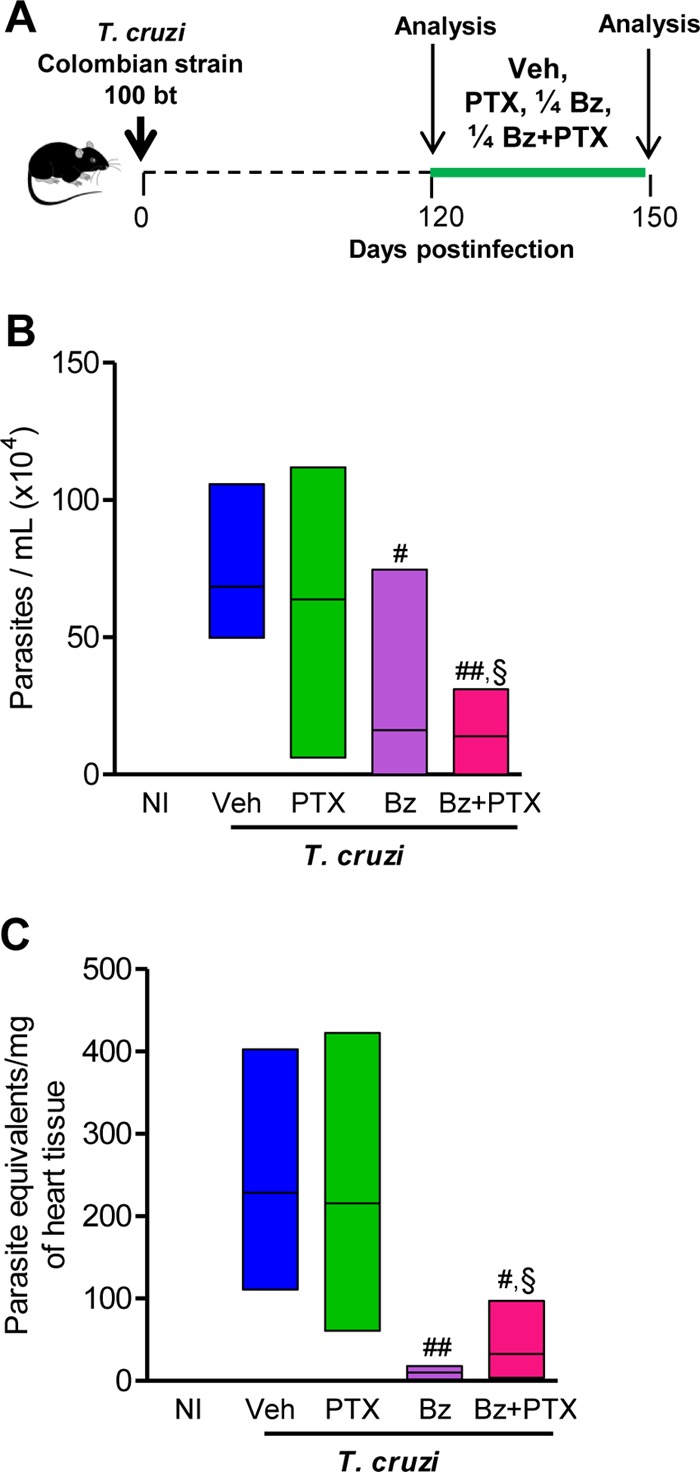
A suboptimal dose of Bz is efficient to control parasite burden, and PTX therapy does not disrupt the efficacy of Bz. (A) Chronically T. cruzi-infected C57BL/6 mice were treated daily (120 to 150 dpi) with Veh, PTX (20 mg/kg in saline, intraperitoneally), or a suboptimal dose of Bz (one-quarter dose of 25 mg/kg in water, by gavage) alone or combined with PTX (Bz plus PTX) and analyzed at 150 dpi. (B) Parasitemia levels. (C) Group data for qPCR detection of T. cruzi kDNA (330 bp). In each experiment, groups consisted of 3 NI controls and 4 or 5 randomly sorted infected mice per experimental group. Mice administered therapies with PTX, Bz, or Bz plus PTX were compared with Veh-injected mice (#, *P* < 0.05; ##, *P* < 0.01). Bz-plus-PTX-treated mice were compared with PTX-treated mice (§, *P* < 0.05). Low-high bar graphs show lines at mean, minimum, and maximum values.

**FIG 2:**
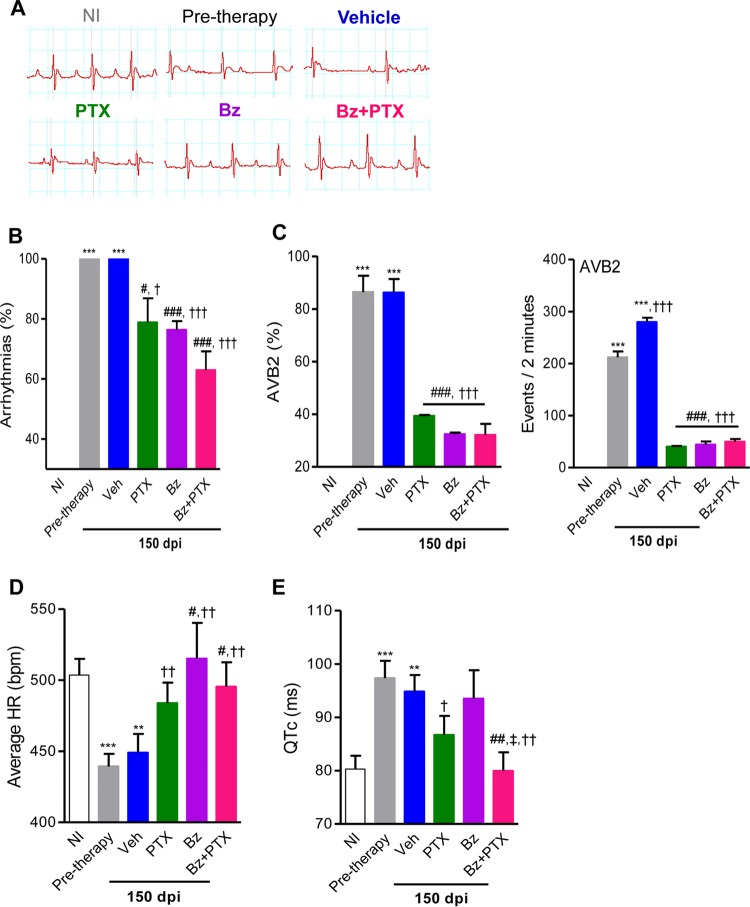
Beneficial effects of PTX, Bz, and Bz-plus-PTX therapies on electrical alterations in experimental Chagas' heart disease. Chronically T. cruzi-infected C57BL/6 mice were treated daily (120 to 150 dpi) with Veh, PTX (20 mg/kg in saline, intraperitoneally), or a suboptimal dose of Bz (one-quarter dose of 25 mg/kg in water, by gavage) alone or combined with PTX (Bz plus PTX) and analyzed at 150 dpi. (A) Representative electrocardiographic register segments of NI controls and infected mice pretherapy (120 dpi) injected with vehicle or administered PTX, Bz, or Bz-plus-PTX therapy and analyzed at 150 dpi. (B) Summary of the group data showing the percentages of mice afflicted by arrhythmias. (C) Group data showing the percentages of mice afflicted by AVB2 and the numbers of AVB2 events recorded in 2 min. (D) Average heart rate (beats per minute). (E) Group data showing QTc interval (milliseconds). Each group consisted of 7 to 10 mice per experiment. Pretherapy and Veh-injected infected mice were compared with NI controls (**, *P* < 0.01; ***, *P* < 0.001). Mice administered therapy with PTX, Bz, or Bz plus PTX were compared with Veh-injected mice (#, *P* < 0.05; ##, *P* < 0.01; ###, *P* < 0.001). Mice treated with Bz plus PTX were compared with Bz-treated mice (‡, *P* < 0.05). Mice treated with PTX, Bz, or Bz plus PTX were compared with pretherapy mice (†, *P* < 0.05; ††, *P* < 0.01; †††, *P* < 0.001). A continuous line over the bars indicates identical statistical results in the three analyzed groups. Graphs show means ± SE.

**FIG 3:**
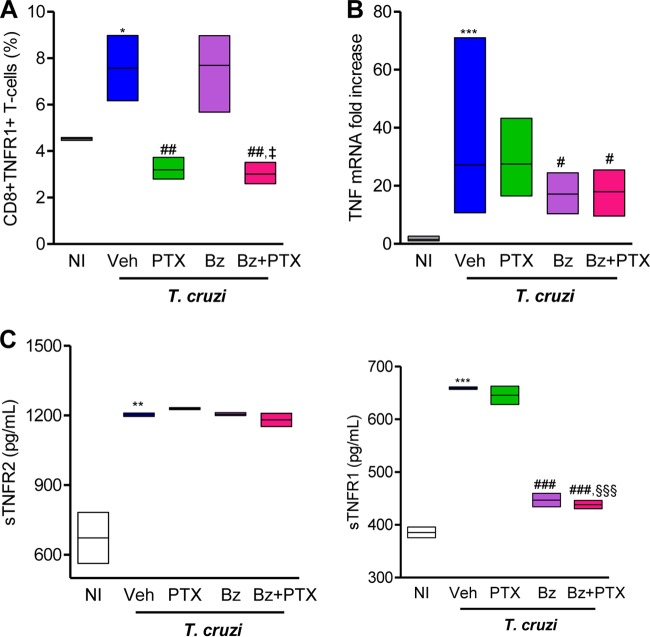
Bz-plus-PTX therapy decreases immune response abnormalities systemically and in heart tissue. Chronically T. cruzi-infected C57BL/6 mice were treated daily (120 to 150 dpi) with Veh, PTX (20 mg/kg, intraperitoneally), or a suboptimal dose of Bz (one-quarter dose of 25 mg/kg, by gavage) alone or combined with PTX (Bz plus PTX) and analyzed at 150 dpi. (A) Frequencies of CD8^+^ TNFR1^+^ T cells in spleen. (B) RT-qPCR for detection of TNF mRNA in heart tissue. (C) ELISA for detection of sTNFR1 and sTNFR2 concentrations in serum. The data represent results from two independent experiments. In each experiment, groups consisted of 3 to 5 randomly sorted mice per group. Veh-injected infected mice were compared with NI controls (*, *P* < 0.05; **, *P* < 0.01; ***, *P* < 0.001). Mice administered PTX, Bz, or Bz plus PTX were compared with Veh-injected mice (#, *P* < 0.05; ##, *P* < 0.01; ###, *P* < 0.001). Mice treated with Bz plus PTX were compared with PTX-treated mice (§§§, *P* < 0.001). Mice treated with Bz plus PTX were compared with Bz-treated mice (‡, *P* < 0.05). Low-high bar graphs show lines at mean, minimum, and maximum values.

Mice obtained from the animal facilities of the Oswaldo Cruz Foundation (CECAL/Fiocruz, Rio de Janeiro, Brazil) were housed under specific-pathogen-free, standard conditions (with temperature and relative humidity of ∼22°C ± 2°C and 55% ± 10%, respectively) in a 12-h light-dark cycle with access to food and water *ad libitum*. The Colombian strain (DTU; TcI strain) is maintained by serial passages from mouse to mouse (C57BL/6) every 35 to 45 days in the Laboratory of Biology of the Interactions, IOC/Fiocruz. For the present study, 5- to 7-week-old female C57BL/6 (H-2^b^) mice were intraperitoneally infected with 100 blood trypomastigotes (bt) of the Colombian strain in 0.2 ml of vaccine-grade sterile buffered saline (BioManguinhos/Fiocruz, Brazil). Parasitemia was estimated by using 5 μl of blood obtained from the tail vein and was employed as a parameter to establish acute and chronic phases (3). The first circulating parasites were detected in blood at 14 days postinfection (dpi), marking the onset of the acute phase of infection. All mice presented peak parasitemia at between 42 and 45 dpi, and trypomastigotes were rarely found in blood at 90 dpi, characterizing the onset of the chronic phase of infection, as previously described (3, 18). On the day of infection, sex- and age-matched noninfected (NI) controls received 0.2 ml of vaccine-grade saline intraperitoneally. In three independent experiments, groups were composed of 22 NI mice and 175 infected mice. The groups were formed as follows. (i) NI controls (total of 22 mice [groups of 7, 7, and 8 mice]) were analyzed by electrocardiogram (ECG) at 120 dpi and 150 dpi. For all analyzed parameters, there were no differences in this group of mice; thus, control mice were combined into one group named “NI,” as shown in Fig. 2. (ii) Infected mice were infected as previously described (3, 13), and 80% of the infected mice survived acute infection (140/175 mice), without trypanocidal treatment, and developed a chronic infection. At 120 dpi, infected mice were randomized to compose a pretherapy group (28/140 mice [groups of 8, 10, and 10 mice]) and treated groups (112/140 mice), which received vehicle (Veh) (34/140 mice [groups of 8, 11, and 15 mice]), PTX (23 mice [groups of 7, 8, and 8 mice]), Bz (28 mice [groups of 8, 10, and 10 mice]), or Bz plus PTX (27 mice [groups of 7, 10, and 10 mice]). At 150 dpi, all infected mice (78/112) treated with PTX, Bz, or Bz plus PTX were alive, while ∼80% of Veh-injected mice survived (27/34 mice). In two independent experiments, at 150 dpi, 3 NI mice and 4 to 6 infected mice were randomly sorted, sacrificed, and analyzed for parasitism; expression of iNOS/NOS2 in heart tissue; and the dosages of nitrite (NO_x_), soluble TNFR1 (sTNFR1), and sTNFR2 in serum, as shown in Fig. 1, 3, and 4. The other NI controls and infected mice were analyzed from 180 to 200 dpi, as shown in Fig. 5.

### Drug treatments.

Chronically T. cruzi-infected C57BL/6 mice showing signs of CCC (3, 22) were treated daily with a suboptimal dose of Bz (one-quarter Bz dose of 25 mg/kg of body weight/day by gavage; Lafepe, Brazil), with or without PTX (20 mg/kg, intraperitoneally) (Trental; Sanofi Aventis, Brazil), from 120 to 150 dpi. Vaccine-grade water and saline were used for dilution of Bz and PTX, respectively, and as Veh controls (BioManguinhos/Fiocruz, Brazil).

### Reagents and antibodies.

For immunohistochemical staining, the polyclonal anti-T. cruzi antibody and supernatants containing anti-mouse CD8a (clone 53-6.7) and anti-mouse CD4 (clone GK1.5) were produced in our laboratory (LBI/IOC-Fiocruz, Rio de Janeiro, RJ, Brazil). Other antibodies included the marker of macrophages anti-F4/80 polyclonal antibody (Caltag, USA), purified goat anti-TNFR1/p55/CD120a (R&D Systems, USA), a biotinylated rabbit anti-goat IgG cocktail (KPL, USA), rabbit antifibronectin (anti-FN; Gibco-BRL, USA), a polyclonal anti-iNOS/NOS2 antibody (Cayman Chemical, USA), biotinylated anti-rat immunoglobulin (Dako, Denmark), and biotinylated anti-rabbit immunoglobulin and a peroxidase-streptavidin complex (both from Amersham, United Kingdom). For flow cytometry studies, phycoerythrin (PE)-Cy7-conjugated anti-mouse T-cell receptor αβ (TCRαβ) (clone H57-597) and allophycocyanin (APC)-conjugated anti-mouse CD8a (clone 53-6.7) were purchased from BD Pharmingen (San Diego, CA, USA), anti-TNFR1 (TNFR1/p55/CD120a; clone 55R-286) conjugated to PE was purchased from BioLegend. Appropriate controls were prepared by replacing the primary antibodies with the corresponding serum or purified immunoglobulin. All antibodies and reagents were used according to the manufacturers' instructions.

### Immunohistochemistry.

Mice were euthanized under anesthesia, and the hearts were removed, embedded in tissue-freezing medium (Tissue-Tek; Miles Laboratories, USA), and stored in liquid nitrogen. T. cruzi parasitism and inflammatory cells expressing CD4, CD8, F4/80, and iNOS/NOS2 were characterized and analyzed as previously described (3, 12, 18). The FN^+^ and iNOS/NOS2^+^ areas in 25 fields (12.5 mm^2^) per section (three sections per heart) were evaluated with a digital morphometric apparatus. The images were digitized by using a color-view XS digital video camera adapted to a Zeiss microscope and analyzed with AnalySIS AUTO software (Soft Imaging System, USA). According to the analyzed parameters, the data are shown as the percent positive area in the heart, distance (micrometers) between stained gap junctions, or numbers of parasite nests or cells per 100 microscopic fields (magnification, ×400).

### Flow cytometry analysis.

Spleens were minced, and red blood cells were removed by using lysis buffer (Sigma-Aldrich, USA). The splenocytes were labeled, events were acquired with a CyAn-ADP instrument (Beckman Coulter, USA), and the data were analyzed with the Summit v.4.3 Build 2445 program (Dako, USA) as described previously (18).

### Real-time quantitative PCR for TNF mRNA.

For real-time quantitative PCR (RT-qPCR), hearts were harvested, washed to remove blood clots, weighed, and frozen in RNAlater (Life Technologies, USA). Total RNA (for gene expression studies) and DNA (for parasite detection/quantification) were extracted from the same sample by using Tri reagent (Sigma-Aldrich, USA), according to the manufacturer's instructions. All reverse transcriptase reactions were performed by using a SuperScript III first-strand synthesis kit, and RT-qPCR was performed by using TaqMan gene expression assays for TNF (catalog number Mm00443258-m1) and the endogenous housekeeping control genes glyceraldehyde-3-phosphate dehydrogenase (GAPDH) (catalog number Mm99999915-g1) and β-actin (catalog number Mm00607939-s1), which were purchased from Life Technologies. Reactions were performed in duplicate according to the manufacturer's instructions, using a cDNA template obtained from 2 μg RNA. The conditions for PCR were as follows: 95°C for 10 min followed by 40 cycles at 95°C for 15 s and 60°C for 1 min. Relative quantification of target gene levels was performed by using the comparative threshold cycle (*C_T_*) (ΔΔ*C_T_*) method (23). RT-qPCR data were normalized by mRNA levels of the housekeeping genes GAPDH and β-actin by using Expression Suite software V1.0.3 (Life Technologies, USA), and fold increases were determined by comparison with NI controls.

### Real-time quantitative PCR for parasite quantification.

Five microliters of purified DNA was analyzed by RT-qPCR using the TaqMan system with primers Cruzi 1 (5′-AST CGG CTG ATC GTT TTC GA-3′) and Cruzi 2 (5′-AAT TCC TCC AAG CAG CGG ATA-3′) and probe Cruzi 3 (6-carboxyfluorescein [FAM]-CACACACTGGACACCAA-MGB) targeting T. cruzi nuclear satellite DNA, as previously described (24). As an internal amplification control, the TaqMan assay targeting mouse GAPDH (catalog number Mm99999915-g1; Life Technologies) was used. The parasite load was estimated by absolute quantification following normalization by heart sample weight. A standard curve was generated by a 1:10 serial dilution of DNA extracted from epimastigote culture stocks of the T. cruzi Colombian strain, ranging from 10^6^ to 0.5 parasite equivalents.

### Measurement of soluble TNF receptor levels.

Enzyme-linked immunosorbent assay (ELISA) kits from R&D Systems were used for the detection of sTNFR1 (catalog number DY425) and sTNFR2 (catalog number DY426) in serum, according to the manufacturer's instructions.

### Quantification of NO concentrations.

Nitrate and NO_x_ levels in serum samples were determined by using the Griess reagent and vanadium chloride III. A standard curve of 0.8 to 100 μM NaNO_2_ and NaNO_3_ was prepared as described previously (12).

### ECG registers.

Mice were intraperitoneally tranquilized with diazepam (10 mg/kg), and transducers were placed subcutaneously according to the chosen preferential derivation (DII). The traces were recorded for 2 min by using a digital Power Lab 2/20 system connected to a bioamplifier at 2 mV for 1 s (PanLab Instruments, Spain). The filters were standardized to between 0.1 and 100 Hz, and the traces were analyzed by using Scope software for Windows V3.6.10 (PanLab Instruments, Spain). We measured heart rate (beats per minute [bpm]); duration of the P wave; and QRS, PR, and QT intervals in milliseconds. The relationship between the QT interval and the RR interval in mouse was assessed for all animals. To obtain physiologically relevant values for the heart rate-corrected QT interval (QTc) in units of time (rather than time to a power that is not equal to 1), the observed RR interval (RR0) was first expressed as a unitless multiple of 100 ms, yielding a normalized RR interval, RR100 = RR0/100 ms. Next, the value of the exponent (*y*) in the relationship QT0 = QTc × RR*y*100 was assessed, with QT0 indicating the observed QT (in milliseconds) and the unit for QTc being milliseconds. The natural logarithm was computed for each side of this relationship [(QT0) = ln(QTc) + *y*ln(RR100)]. Thus, the slope of the linear relationship between the log-transformed QT and RR100 defined the exponent to which the RR interval ratio should be raised to correct QT for heart rate (3).

### Statistical analysis.

Data are expressed as means ± standard errors (SE). Analysis was performed by using GraphPad Prism (GraphPad, USA). Comparison between groups was carried out by analysis of variance (ANOVA) followed by Bonferroni's posttest or Fisher's exact test, when indicated. The Kaplan-Meier test was used to compare the survival times of the studied groups. Differences were considered statistically significant when the *P* value was <0.05.

## RESULTS

### Bz and Bz-plus-PTX administrations reduce parasite load.

In Fig. 1A, the experimental design shows that C57BL/6 mice infected with the Colombian T. cruzi strain were treated with Veh, PTX (20 mg/kg/day), and a suboptimal dose of Bz (one-quarter dose of 25 mg/kg/day) alone or combined with PTX (Bz plus PTX) during 30 consecutive days. Drug administration started at 120 dpi, when electrical abnormalities and heart injury are already detected (3, 22). At 150 dpi, all infected mice treated with PTX (23/23 mice; 100% ± 0%), Bz (28/28; 100% ± 0%), or Bz plus PTX (27/27; 100% ± 0%) were alive, while ∼80% (27/34; 79% ± 2%) of Veh-injected mice survived (*P* < 0.05). Initially, we tested the impacts of these therapeutic schemes on parasitemia and heart parasitism. At 150 dpi, compared with injection of Veh (68 ± 9 parasites/ml), PTX treatment (64 ± 23 parasites/ml) did not alter the number of circulating parasites. However, in mice treated with Bz (16 ± 15 parasites/ml; *P* < 0.05) and Bz plus PTX (14 ± 8 parasites/ml; *P* < 0.01), parasitemia was reduced (Fig. 1B). In comparison with PTX treatment, the combination therapy significantly reduced the number of circulating parasites (*P* < 0.05 for PTX versus Bz plus PTX). In addition, in combination therapy, PTX did not abrogate the beneficial effects of Bz on decreasing parasitemia (*P* > 0.05 for Bz versus Bz plus PTX). Furthermore, using real-time quantitative PCR, a high-sensitivity method to detect specific parasite DNA sequences (25), we showed that in comparison to Veh-injected mice (229 ± 69 parasite equivalents/mg of tissue), there was no alteration in parasite burden in the cardiac tissues of PTX-treated mice (216 ± 64 parasite equivalents/mg of tissue). However, in comparison to injection of Veh, the parasite load was significantly reduced in heart tissue after Bz (10 ± 3 parasite equivalents/mg of heart tissue; *P* < 0.01) and Bz-plus-PTX (32 ± 22 parasite equivalents/mg of tissue; *P* < 0.05) therapies (Fig. 1C). In comparison to PTX treatment, combination therapy also reduced parasite loads in the cardiac tissue (*P* < 0.05 for PTX versus Bz plus PTX) (Fig. 1C). Again, in combination therapy, PTX did not abrogate the beneficial effects of Bz (*P* > 0.05 for Bz versus Bz plus PTX). Notably, in chronic infection with the Colombian strain, the suboptimal dose of Bz was efficient in reducing parasite burden in the peripheral blood and in heart tissue. Moreover, PTX administration in association with Bz did not limit Bz efficacy in the periphery and in heart tissue (Fig. 1B and C).

### Combination therapy of Bz plus PTX restores normal QTc intervals.

Electrical abnormalities were not observed for all NI control mice (0/22 mice) analyzed in parallel with T. cruzi-infected mice at 120 dpi and 150 dpi; therefore, the data are shown as results for one group (NI). Representative ECG recordings show that pretherapy (120 dpi) and Veh-injected (150 dpi) infected mice presented electrical abnormalities compared with NI controls. The three therapeutic schemes ameliorated the electrical abnormalities (Fig. 2A). Relevant electrical abnormalities detected in patients with Chagas' heart disease, such as arrhythmia (ART), second-degree atrioventricular block (AVB2), bradycardia, and a prolonged corrected QT (QTc) interval (26), were reproduced in Colombian strain-infected C57BL/6 mice (Fig. 2B to E). In three independent experiments, at 120 dpi (pretherapy), all infected mice presented ART (28/28 mice; 100% ± 0%), and the majority of them showed AVB2 (24/28 mice; 86% ± 6%), with elevated numbers of AVB2 events being registered in 2-min recordings (212 ± 11 events), as shown in Fig. 2B and C. At 150 dpi, as shown in Fig. 2B and C, Veh-injected T. cruzi-infected C57BL/6 mice presented ART (27/27 mice; 100% ± 0%) and AVB2 (23/27 mice; 86% ± 5%). Furthermore, in comparison with mice at 120 dpi, Veh-injected infected mice showed elevated numbers of AVB2 events registered in 2-min recordings (280 ± 8 events; *P* < 0.001) (Fig. 2C). At 150 dpi, compared with vehicle administration, PTX (17/23 mice [78% ± 8%]; *P* < 0.05), Bz (21/28 mice [76% ± 3%]; *P* < 0.001), and Bz-plus-PTX (16/27 mice [63% ± 6%]; *P* < 0.001) treatments reduced the percentages of mice afflicted by ART (Fig. 2B). Furthermore, the three therapeutic schemes reduced the percentage of mice with AVB2 and the number of AVB2 events in 2-min recordings (9/23 mice [39% ± 0.4%], 40 ± 1.4 events, and *P* < 0.001 for PTX; 9/28 mice [32% ± 0.6%], 44 ± 2 events, and *P* < 0.001 for Bz; and 8/27 mice [32% ± 4%], 50 ± 1.7 events, and *P* < 0.001 for Bz plus PTX), as shown in Fig. 2C.

Analysis of heart rate (beats per minute) showed that in comparison with NI controls (504 ± 11 bpm), pretherapy (120 dpi) infected mice (439 ± 9 bpm; *P* < 0.001) and Veh-injected infected mice (449 ± 13 bpm; *P* < 0.01) presented bradycardia (Fig. 2D). At 150 dpi, in comparison with injection of Veh, treatment with PTX had no significant effect (484 ± 14 bpm; *P* > 0.05), while therapeutic schemes using Bz (515 ± 25 bpm; *P* < 0.05) and Bz plus PTX (495 ± 17 bpm; *P* < 0.05) significantly improved heart rates (Fig. 2D). Furthermore, compared with NI controls (80.3 ± 2.5 ms), infected mice at 120 dpi (97.3 ± 3.2 ms; *P* < 0.001) and Veh-injected T. cruzi-infected mice at 150 dpi (94.8 ± 3 ms; *P* < 0.01) presented prolonged QTc intervals (Fig. 2E). At 150 dpi, compared with injection of Veh, treatments with PTX (86.7 ± 3.6 ms; *P* > 0.05) and Bz (93.5 ± 5.3 ms; *P* > 0.05) had no effect; however, Bz-plus-PTX therapy (80 ± 3.5 ms; *P* < 0.01) ameliorated the prolonged QTc interval of chronically infected mice (Fig. 2E).

Altogether, in comparison with pretherapy infected mice (120 dpi), the three therapeutic schemes were beneficial and reversed the numbers of mice afflicted by ART and AVB2 (Fig. 2B) and significantly reduced the numbers of AVB2 events registered in 2-min intervals (Fig. 2C). In addition, the three schemes reversed bradycardia induced by T. cruzi infection (Fig. 2D). Although Bz had no effect (*P* > 0.05), PTX therapy (*P* < 0.05) partially reversed the prolonged QTc interval induced by T. cruzi infection. Notably, in comparison with pretherapy mice, Bz plus PTX completely reversed the prolonged QTc interval (*P* < 0.01) and restored QTc intervals resembling those of NI mice (Fig. 2E). Thus, considering ECG analysis, Bz-plus-PTX therapy was more favorable than the other therapeutic schemes used in chronically T. cruzi-infected mice (Fig. 2). In summary, in comparison with PTX and Bz single-drug therapies, Bz-plus-PTX combination therapy simultaneously ameliorated parasite burden and electrical abnormalities in chronically T. cruzi-infected mice (Table 1).

**TABLE 1:** Combination therapy with PTX plus Bz ameliorates parasite control and electrical abnormalities in chronically T. cruzi-infected mice^*a*^

Parameter	Mean value for treatment group ± SE					
	NI	Pretherapy (120 dpi)	Posttherapy (150 dpi)			
			Veh	PTX	Bz	Bz+PTX
Parasitemia level (10^4^ parasites/ml)	0	53 ± 10	68 ± 9 ††	64 ± 23 #	16 ± 15 #	14 ± 8 ##,§
Heart parasite load (parasite equivalents/mg)	0	NT	229 ± 69	216 ± 64	10 ± 3 ##	32 ± 22 #,§
% of mice with arrhythmia	0	100 ± 0 ***	100 ± 0 ***	78 ± 8 #,†	76 ± 3 ###,†††	63 ± 6 ###,†††
No. of flutter events/2 min	0	0	38.5 ± 2.1 ***,†††	0 ###	0 ###	0 ###
% of mice with AVB2	0	86 ± 6 ***	86 ± 5 ***	39 ± 0.4 ###,†††	32 ± 0.6 ###,†††	32 ± 4 ###,†††
No. of AVB2 events/2 min	0	212 ± 11 ***	280 ± 8 ***,†††	40 ± 1.4 ###,†††	44 ± 2 ###,†††	50 ± 1.7 ###,†††
Avg HR (bpm)	504 ± 11	439 ± 9 ***	449 ± 13 **	484 ± 14 ††	515 ± 25 #,††	495 ± 17 #,††
QTc (ms)	80 ± 2.5	97.3 ± 3.2 ***	94.8 ± 3 **	86.7 ± 3.6 †	93.5 ± 5.3	79.9 ± 3.5 ##,‡,††

a NT, not tested; HR, heart rate. *, *P* < 0.05; **, *P* < 0.01; ***, *P* < 0.001 (for comparison of vehicle-infected T. cruzi-infected mice with NI mice). #, *P* < 0.05; ##, *P* < 0.01; ###, *P* < 0.001 (for comparison of T. cruzi-infected mice administered PTX, Bz, or Bz-plus-PTX treatment with Veh-injected infected mice). §, *P* < 0.05 (for comparison of T. cruzi-infected mice administered Bz-plus-PTX treatment with infected mice treated with PTX). ‡, *P* < 0.05 (for comparison of T. cruzi-infected mice administered Bz-plus-PTX treatment with infected mice treated with Bz). †, *P* < 0.05; ††, *P* < 0.01; †††, *P* < 0.001 (for comparison of T. cruzi-infected mice administered PTX, Bz, or Bz-plus-PTX treatment with infected mice pretherapy [120 dpi]).

### Chronically infected mice treated with Bz plus PTX are benefited by individual effects of Bz and PTX on TNF and TNFR1 expression.

In chronically infected mice, PTX therapy reduced TNFR1/p55 upregulation on CD8^+^ T cells but did not affect TNF mRNA overexpression in heart tissue (18). On the other hand, Bz was shown to attenuate sepsis-induced TNF overproduction (27). Therefore, we examined the expression of TNF and its receptors (TNFR1/p55 and TNFR2/p75) after the use of the proposed therapeutic schemes. At 150 dpi, compared with age-matched NI controls (4.5% ± 0.04%), there was a significant increase in the frequency of CD8^+^ T cells expressing TNFR1 in Veh-injected mice (7.6% ± 0.8%; *P* < 0.05). Although Bz treatment had no effect (7.7% ± 1%; *P* > 0.05), PTX (3.2 ± 0.3%; *P* < 0.01) and Bz-plus-PTX (3.0% ± 0.3%; *P* < 0.01) administrations reduced the frequency of CD8^+^ TNFR1^+^ T cells in the spleens of chronically infected mice (Fig. 3A). In addition, a significant reduction in the frequency of CD8^+^ TNFR1^+^ T cells was observed in the spleens of Bz-plus-PTX-treated mice in comparison to Bz-treated mice (*P* < 0.05) (Fig. 3A).

At 150 dpi, in comparison with NI controls, there was an increase in the TNF mRNA expression level in heart tissue of Veh-injected infected mice (27-fold ± 7-fold increase; *P* < 0.05). This augmented expression of TNF mRNA was not impacted by PTX treatment (27-fold ± 5-fold increase; *P* > 0.05) but was decreased by Bz (17-fold ± 2-fold increase; *P* < 0.01) and Bz-plus-PTX (17-fold ± 3-fold increase; *P* < 0.01) therapies (Fig. 3B).

We also examined the expression levels of soluble TNFRs, which are implicated in physiological and pathological cardiac conditions (28, 29). At 150 dpi, the high plasma sTNFR2 concentrations found in infected mice (673 ± 110 pg/ml in NI controls versus 1,203 ± 7 pg/ml in Veh-injected mice; *P* < 0.01) remained elevated after all treatments were administered (1,229 ± 4 pg/ml in PTX-treated mice, 1,206 ± 6 pg/ml in Bz-treated mice, and 1,181 ± 28 pg/ml in Bz-plus-PTX-treated mice; *P* > 0.05), as shown in Fig. 3C. In comparison with NI controls (386 ± 10 pg/ml), Veh-injected infected mice (659 ± 2.4 pg/ml; *P* < 0.001) showed increased concentrations of sTNFR1. In comparison with Veh-injected mice, elevated levels of sTNFR1 were also detected in the sera of PTX-treated infected mice (645 ± 17 pg/ml; *P* > 0.05). However, there was a significant reduction in sTNFR1 concentrations in the sera of mice treated with Bz (447 ± 13 pg/ml; *P* < 0.001) and Bz-plus-PTX (438 ± 7.8 pg/ml; *P* < 0.001). Furthermore, a significant reduction in the sTNFR1 concentration was detected in the sera of Bz-plus-PTX-treated compared with PTX-treated mice (*P* < 0.001) (Fig. 3C).

### Therapeutic schemes ameliorate fibrosis and inflammation in heart tissue.

Fibrosis is a remarkable feature of Chagas' heart disease (30). Compared with NI controls, chronically Colombian strain-infected C57BL/6 mice exhibited FN overdeposition in heart tissue (17% ± 0.5% FN^+^ area in NI controls versus 43% ± 1.5% FN^+^ area in Veh-injected infected mice; *P* < 0.001). Interestingly, FN overdeposition was reduced after PTX (25.3% ± 1.2% FN^+^ area; *P* < 0.001), Bz (15.7% ± 1.1% FN^+^ area; *P* < 0.001), and Bz-plus-PTX (22.3% ± 1.7% FN^+^ area; *P* < 0.001) treatments (Table 2).

**TABLE 2:** Therapeutic schemes ameliorate fibrosis and inflammation in heart tissues of chronically T. cruzi-infected mice

Parameter	Mean value for treatment group ± SE^*a*^				
	NI	Veh	PTX	Bz	Bz+PTX
FN^+^ area (%)	17 ± 0.5	43 ± 1.5 ***	25.3 ± 1.2 ###	15.7 ± 1.1 ###	22.3 ± 1.7 ###
Inflammation (no. of cells/100 fields)	0.5 ± 0.3	1,258 ± 138 ***	816 ± 44 #	517 ± 67 #	578 ± 124 #

a ***, *P* < 0.001 (for comparison of vehicle-treated *T. cruzi*-infected mice with NI mice). #, *P* < 0.05; ###, *P* < 0.001 (for comparison of T. cruzi-infected mice administered PTX, Bz, or Bz-plus-PTX treatment with Veh-injected infected mice).

Another important feature of CCC is low-grade myocarditis (30). As shown in Table 2, in comparison with NI controls (0.5 ± 0.3 cells/100 microscopic fields), chronically Colombian strain-infected C57BL/6 mice injected with vehicle (1,258 ± 138 cells/100 microscopic fields; *P* < 0.001) presented heart tissue inflammation. Compared with vehicle-injected, chronically infected mice treated with PTX (816 ± 44 cells/100 microscopic fields; *P* < 0.05), Bz (517 ± 67 cells/100 microscopic fields; *P* < 0.05) and Bz plus PTX (578 ± 124 cells/100 microscopic fields; *P* < 0.05) showed reduced numbers of mononuclear cells (composed of macrophages, CD4^+^ cells, and, mainly, CD8^+^ cells) infiltrating the heart tissue.

### PTX increases the effects of Bz on reduction of iNOS/NOS2 expression levels in heart tissue and NO concentrations in the serum.

**FIG 4:**
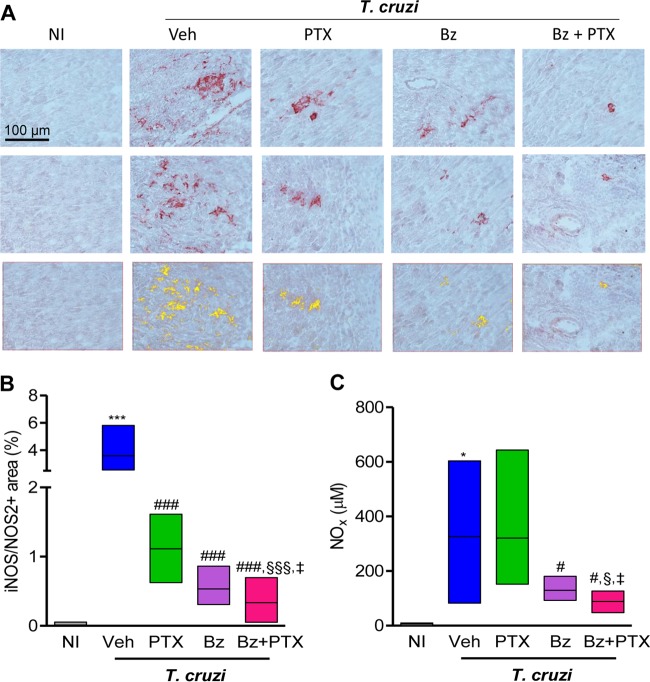
PTX potentiates the effect of Bz on the reduction of iNOS/NOS2 expression levels in heart tissue and NO_x_ concentrations in serum. Chronically T. cruzi-infected C57BL/6 mice were treated daily (120 to 150 dpi) with Veh, PTX (20 mg/kg, intraperitoneally), or a suboptimal dose of Bz (one-quarter dose of 25 mg/kg, by gavage) alone or combined with PTX (Bz plus PTX) and analyzed at 150 dpi. (A, top and middle) Representative heart sections from each experimental group analyzed by immunohistochemical staining to detect iNOS/NOS2. (Bottom) Representative images used to analyze the percent iNOS/NOS2^+^ area by using AnalySIS AUTO software. (B) Quantification of the percent iNOS/NOS2^+^ area in heart tissue. (C) Concentrations of NO_x_ in serum. The data represent results from two independent experiments. In each experiment, groups consisted of 3 NI controls and 3 to 6 randomly sorted mice. Veh-injected infected mice were compared with NI controls (*, *P* < 0.05; ***, *P* < 0.001). Mice administered PTX, Bz, or Bz plus PTX were compared with Veh-injected mice (#, *P* < 0.05; ###, *P* < 0.001). Mice treated with Bz plus PTX were compared with PTX-treated mice (§, *P* < 0.05; §§§, *P* < 0.001). Mice treated with Bz plus PTX were compared with Bz-treated mice (‡, *P* < 0.05). Low-high bar graphs show lines at mean, minimum, and maximum values.

The participation of iNOS/NOS2 and NO in CCC severity has been proposed (6, 7, 12, 13, 22). At 150 dpi, compared with age-matched NI controls, chronically T. cruzi-infected mice showed increased iNOS/NOS2 expression levels in cells morphologically compatible with cardiomyocytes, endothelial cells, and inflammatory cells infiltrating the heart tissue (Fig. 4A). In comparison with NI controls (0.01% ± 0.01% iNOS^+^ area), Veh-injected infected mice (3.6% ± 0.3% iNOS^+^ area; *P* < 0.001) presented increased percentages of iNOS/NOS2^+^ areas in heart tissue (Fig. 4B). Furthermore, a significant reduction in the percentage of the iNOS/NOS2^+^ area was detected after PTX (1.1% ± 0.13% iNOS^+^ area; *P* < 0.001) and Bz (0.53% ± 0.08% iNOS^+^ area; *P* < 0.001) therapies (Fig. 4A and B). Notably, a more pronounced reduction of iNOS/NOS2^+^ areas in the cardiac tissue was observed in Bz-plus-PTX-treated mice (0.33% ± 0.06% iNOS^+^ area) than in Veh-injected (*P* < 0.001), PTX-treated (*P* < 0.001), and Bz-treated (*P* < 0.05) chronically infected mice (Fig. 4B).

At 150 dpi, compared with NI control mice (5.7 ± 2.8 μM), a significant increase in NO_x_ concentrations was observed in the sera of Veh-injected mice (326 ± 80.6 μM; *P* < 0.05), as shown in Fig. 4C. Moreover, in comparison with mice injected with Veh, although PTX therapy (321 ± 89.7 μM) had no effect, Bz (134.9 ± 14.3 μM; *P* < 0.05) and Bz-plus-PTX (88.4 ± 13.7 μM; *P* < 0.05) administrations to infected mice significantly reduced NO_x_ levels in serum (Fig. 4C). Notably, administration of Bz plus PTX to chronically infected mice reduced NO_x_ levels in serum in a more prominent manner than did PTX (*P* < 0.05) or Bz (*P* < 0.05), as shown in Fig. 4C.

### Combination therapy with Bz plus PTX sustains the beneficial effects on parasite control and electrical abnormalities after time-defined administration.

**FIG 5:**
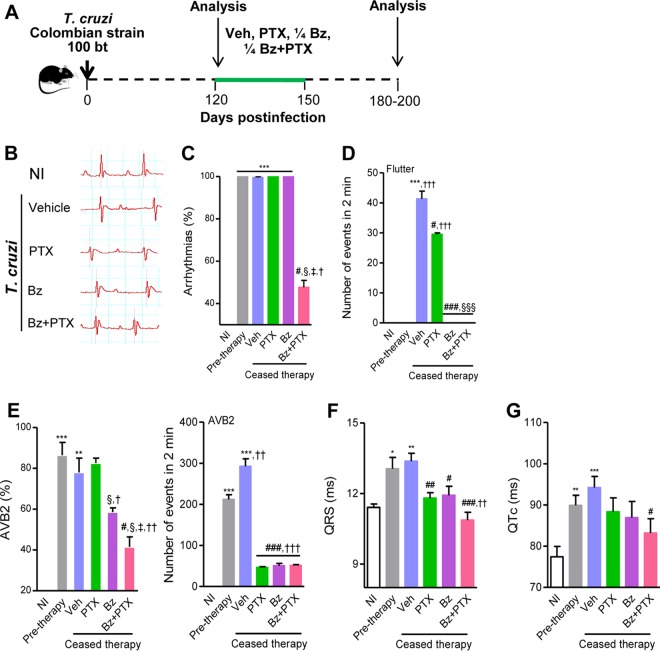
Sustained effect of Bz-plus-PTX therapy on electrical abnormalities after time-defined treatment. (A) Chronically T. cruzi-infected C57BL/6 mice were treated daily (120 to 150 dpi) with Veh, PTX (20 mg/kg, intraperitoneally), or a suboptimal dose of Bz (one-quarter dose of 25 mg/kg, by gavage) alone or combined with PTX (Bz plus PTX) and analyzed 30 to 50 days after therapy cessation (180 to 200 dpi). (B) Representative electrocardiographic register segments of NI controls and infected mice injected with vehicle or administered PTX, Bz, or Bz-plus-PTX therapy. (C) Summary of the group data showing the percentages of mice afflicted by arrhythmias. (D) Numbers of atrial flutter events in registers of 2 min. (E) Summary of the group data showing the percentages of mice afflicted by AVB2 and the numbers of AVB2 events in registers of 2 min. (F) Group data showing QRS intervals (milliseconds). (G) Group data showing QTc intervals (milliseconds). Each group consisted of 6 to 11 mice. Pretherapy and Veh-injected infected mice were compared with NI controls (*, *P* < 0.05; **, *P* < 0.01; ***, *P* < 0.001). Mice administered PTX, Bz, or Bz plus PTX were compared with Veh-injected mice (#, *P* < 0.05; ##, *P* < 0.01; ###, *P* < 0.001). Mice treated with Bz plus PTX were compared with PTX-treated mice (§, *P* < 0.05; §§§, *P* < 0.001). Mice treated with Bz plus PTX were compared with Bz-treated mice (‡, *P* < 0.05). Mice treated with PTX, Bz, or Bz plus PTX were compared with pretherapy mice (†, *P* < 0.05; ††, *P* < 0.01; †††, *P* < 0.001). A continuous line over the bars indicates identical statistical results in the two or three analyzed groups. Graphs show means ± SE.

In chronically T. cruzi-infected mice, Bz-plus-PTX therapy ameliorates electrical abnormalities, particularly reducing the prolonged QTc interval (Fig. 2). Furthermore, in Bz-plus-PTX-treated mice, we detected complementary beneficial effects of Bz and PTX on abnormalities of the immune response (Fig. 3 and 4), which were previously associated with ECG changes (9, 18). Therefore, we predicted that the favorable effects of Bz-plus-PTX therapy on ECG alterations would persist after therapy discontinuation. To test this idea, chronically infected mice were submitted to the therapeutic schemes (120 to 150 dpi) and analyzed 30 to 50 days after treatment cessation (at 180 to 200 dpi), as schematically shown in Fig. 5A. After therapy discontinuation, in comparison with infected mice injected with Veh (56 × 10^4^ ± 45 × 10^4^ parasites/ml), parasite burden was controlled in chronically infected mice treated with PTX (23 × 10^4^ ± 8.5 × 10^4^ parasites/ml; *P* < 0.05), Bz (9.9 × 10^4^ ± 9.9 × 10^4^ parasites/ml; *P* < 0.01), and Bz plus PTX (5.7 × 10^4^ ± 3 × 10^4^ parasites/ml; *P* < 0.001).

At 180 to 200 dpi, 30 to 50 days after therapy cessation, all Veh-injected, PTX-treated, and Bz-treated chronically infected mice presented ART (Fig. 5B and C), showing that the beneficial effects of PTX and Bz observed at 150 dpi were transient. Conversely, the amelioration of ART was sustained in infected mice subjected to Bz-plus-PTX (48% ± 3%; *P* < 0.05) combination therapy (Fig. 5B and C). At 120 dpi (pretherapy), atrial flutter was not detected in T. cruzi-infected mice. (Fig. 5D). At 150 dpi, atrial flutter was detected in Veh-injected mice (15% of mice; 38.5 ± 2.1 events in 2-min recordings) but not in mice treated with PTX, Bz, or Bz plus PTX (Table 1). At 180 to 200 dpi (30 to 50 days after therapy cessation), atrial flutter was detected in Veh-injected mice (43% of mice; 41.3 ± 2.6 events in 2-min recordings). Atrial flutter was also present in PTX-treated infected mice, with a reduced number of events (20% of mice; 29.5 ± 0.5 events in 2-min recordings; *P* < 0.05) but was not detected in Bz- and Bz-plus-PTX-treated infected mice and age-matched NI controls (Fig. 5D). Similarly to mice pretherapy (120 dpi), when the majority of T. cruzi-infected animals showed AVB2 (86% ± 6%), Veh-injected mice (at 180 to 200 dpi) presented a high frequency of AVB2 (78% ± 7%). In addition, compared with mice pretherapy, which presented a high number of AVB2 events in 2-min recordings (212 ± 11 events), there was a significant increase in the number of events in Veh-injected mice at 180 to 200 dpi (292 ± 18 events; *P* < 0.001), as shown in Fig. 5E. Furthermore, after therapy cessation, a high proportion of mice treated with PTX showed AVB2 (82% ± 2%), but the numbers of events in 2-min recordings were reduced (47 ± 1.5 events; *P* < 0.001). Notably, compared with mice injected with Veh, there were significant decreases in the percentages of mice afflicted by AVB2 and the numbers of events in 2-min recordings 30 to 50 days after discontinuation of treatments with Bz (58% ± 1.5% [*P* < 0.05]; 50 ± 2.8 events [*P* < 0.001]) and with Bz plus PTX (41% ± 2% [*P* < 0.05]; 51 ± 1.2 events [*P* < 0.001]), as shown in Fig. 5E.

After therapy discontinuation, compared with Veh-injected mice (13.4 ± 0.3 ms), mice administered treatment with PTX (11.8 ± 0.2 ms; *P* < 0.01), Bz (11.9 ± 0.4 ms; *P* < 0.05), or Bz plus PTX (10.9 ± 0.3 ms; *P* < 0.001) showed a reduction in the prolonged QRS interval. Furthermore, in mice treated with Bz plus PTX, the prolonged QRS interval was reversed (*P* < 0.01) in comparison with pretherapy mice and restored to patterns resembling those for NI controls (Fig. 5F). Finally, the beneficial effects of PTX on the prolonged QTc interval (Fig. 2) ceased after therapy interruption (88.3 ± 3.4 ms in PTX-treated mice versus 94.2 ± 2.7 ms in Veh-injected mice), but there was no progression in comparison with pretherapy mice (89.9 ± 2.4 ms), as shown in Fig. 5G. In comparison with pretherapy (120 dpi) and age-matched Veh-injected (180 to 200 dpi) infected mice, Bz-treated mice also showed no progression of prolonged QTc (86.9 ± 3.9 ms). Importantly, compared with Veh-injected mice, Bz-plus-PTX-treated mice presented a reduction in the prolonged QTc interval (83.2 ± 3.4 ms; *P* < 0.05) (Fig. 5G). Altogether, our data support that after therapy interruption, Bz-plus-PTX treatment showed sustained beneficial effects on parasite control and electrical abnormalities such as ART, AVB2, and QRS and QTc intervals (Table 3).

**TABLE 3:** Combination therapy of PTX plus Bz shows sustained beneficial effects on parasite control and electrical abnormalities in chronically T. cruzi-infected mice after treatment cessation

Parameter	Mean value for group ± SE^*a*^					
	NI	Pretherapy (120 dpi)	Posttherapy (180–200 dpi)			
			Veh	PTX	Bz	Bz+PTX
Parasitemia level (10^4^ parasites/ml)	0	53 ± 10	56 ± 45	23 ± 8.5 #	9.9 ± 9.9 ##	5.7 ± 3 ###
% of mice with arrhythmia	0	100 ± 0 ***	100 ± 0 ***	100 ± 0 ***	100 ± 0 ***	48 ± 3 #,§,‡,†
No. of flutter events/2 min	0	0	41.3 ± 2.6 ***,†††	29.5 ± 0.5 #,†††	0 ###,§§§	0 ###,§§§
% of mice with AVB2	0	86 ± 6 ***	78 ± 7 ***	82 ± 2	58 ± 1.5 §,†	41 ± 2 #,§,‡,††
No. of AVB2 events/2 min	0	212 ± 11 ***	292 ± 18 ***,††	47 ± 1.5 ###,†††	50 ± 2.8 ###,†††	51 ± 1.2 ###,†††
QRS (ms)	11 ± 0.14	13 ± 0.48 *	13.4 ± 0.3 ***	11.8 ± 0.2 ##	11.9 ± 0.4 #	10.9 ± 0.3 ###,††
QTc (ms)	77 ± 2.5	89.9 ± 2.4 **	94.2 ± 2.7 ***	88.3 ± 3.4	86.9 ± 3.9	83.2 ± 3.4#

a *, *P* < 0.05; **, *P* < 0.01; ***, *P* < 0.001 (for comparison of vehicle-treated T. cruzi-infected mice with NI mice). #, *P* < 0.05; ##, *P* < 0.01; ###, *P* < 0.001 (for comparison of T. cruzi-infected mice administered PTX, Bz, or Bz-plus-PTX treatment with Veh-injected infected mice). §, *P* < 0.05 (for comparison of T. cruzi-infected mice administered Bz-plus-PTX treatment with infected mice treated with PTX). ‡, *P* < 0.05 (for comparison of T. cruzi-infected mice administered Bz-plus-PTX treatment with infected mice treated with Bz). †, *P* < 0.05; ††, *P* < 0.01; †††, *P* < 0.001 (for comparison of T. cruzi-infected mice administered PTX, Bz, or Bz-plus-PTX treatment with infected mice pretherapy [120 dpi]).

## DISCUSSION

In the present study, we challenged the idea that T. cruzi persistence and parasite-driven immune deregulation contribute to CCC. In addition, we proposed therapeutic schemes based on parasite control (using the trypanocidal drug Bz), repositioning of the immune response (using the immunoregulator PTX), or the combination of both strategies. The suboptimal dose of Bz reduced parasite burden, and PTX did not disrupt Bz efficacy. Although the three therapeutic schemes played a beneficial role in infection-induced electrical abnormalities, the effects of Bz-plus-PTX combination therapy were more prominent. The individual beneficial effects of Bz and PTX on the unbalanced immune response, interfering in TNF/TNFR and iNOS/NO pathways, were detected in a complementary and/or potentialized form in Bz-plus-PTX-treated mice. Moreover, after therapy cessation, Bz plus PTX sustained parasite burden control and reversion of major electrical abnormalities induced by T. cruzi infection.

Consensually, T. cruzi persistence and parasite-driven immune deregulation contribute to CCC (4, 7, 18, 22). Currently, medicaments used to treat T. cruzi infection have limited efficacy and registered toxicity; therefore, there is a need for new drugs with higher efficacy and fewer side effects to address parasite load, reduce immunological abnormalities, and improve clinical signs (14, 15, 31). One possible strategy is combination therapy, as previously used with success for other infections such as tuberculosis, human immunodeficiency virus, and malaria (32–34). A pilot study based on parasite control carried out with chronic CD patients using sequential treatments with allopurinol and Bz revealed decreased CD4^+^ and CD8^+^ T-cell activation, indicative of a reduction in parasite burden (35). In mouse infection, therapy with Bz (100 mg/kg/day) initiated in the acute phase prevented electrical abnormalities in the chronic phase of infection without complete elimination of T. cruzi parasites (20). Side effects and questionable efficacy, especially in the case of resistant T. cruzi strains, have hampered Bz usage in chronic T. cruzi infection (14, 19). In patients with noninfectious ischemic cardiomyopathy, in comparison with placebo, PTX has previously shown cardioprotective effects in association with reductions in plasma concentrations of TNF (16). Furthermore, PTX was beneficial for ECG alterations and hampered T-cell abnormalities in chronically infected mice with signs of CCC and an unbalanced immune response, independently of the persistence of low-grade heart parasitism (18). Therefore, conceptually aiming to reeducate the immune response and to control parasite burden and being aware of the need for minimizing the adverse effects of Bz (31), we combined PTX with a suboptimal dose (25 mg/kg/day) of the trypanocidal drug Bz.

Our data support that therapy with a suboptimal dose of Bz (25 mg/kg/day) reduced the already low parasite load in chronically Colombian strain-infected mice. Also, we corroborated that PTX therapy did not alter blood and heart parasitism (18). Moreover, our data support that PTX administration combined with the suboptimal dose of Bz did not interfere with the trypanocidal efficacy of Bz. Although the parasite load in peripheral blood and heart tissue was reduced after Bz and Bz-plus-PTX therapies, there was no parasitological cure. One can argue that this was due to the resistance of the Colombian strain to Bz; however, our results corroborate previously reported data showing that optimal (100 mg/kg/day) and suboptimal (25 mg/kg/day) Bz doses were efficient to reduce parasite load and promote survival in immunocompetent mice infected with the Colombian T. cruzi strain (20, 36). Nevertheless, parasite elimination is not a prerequisite for better prognosis in CD. A recent study revealed no differences in parasite loads in comparisons of patients with the indeterminate form with patients with the digestive, cardiac, or cardiodigestive form of CD (37). Furthermore, observational clinical studies have shown that chronic CD patients subjected to Bz therapy, although not cured, had a significant reduction in the occurrence of ECG changes and a lower frequency of deterioration of clinical condition (38). However, Bz administered to CD patients with cardiomyopathy played an accessory role in the well-established immunological control of parasite reducing T. cruzi DNA in serum but failed to improve clinical outcomes in a 5-year follow-up study (19). Indeed, Bz administration to chronically infected dogs was efficient for parasite control but did not prevent CCC (39). On the other hand, treatment with Bz (100 mg/kg/day) initiated in the acute phase of infection prevented severe CCC despite the lack of complete parasite eradication (20). Additionally, Bz (100 mg/kg/day) has been shown to prevent and reverse cardiac fibrosis in chronically T. cruzi-infected mice (40). Here, we showed that the suboptimal dose of Bz, besides reducing parasite load, also decreased heart tissue fibrosis. Recently, PTX has been shown to ameliorate electrical and functional cardiac alterations in chronically T. cruzi-infected mice, repositioning CD8^+^ T-cell abnormalities without interfering with parasite load (18). This group of data emphasizes the complexity of the mechanisms contributing to CCC pathogenesis. Therefore, to reduce toxicity and to improve CCC prognosis, we proposed a combination therapy using a suboptimal dose of the trypanocidal drug Bz and the immunoregulator PTX.

The use of therapies with PTX, Bz, and Bz plus PTX in chronically T. cruzi-infected mice with signs of CCC reduced the frequencies of mice presenting ART and AVB2. Furthermore, these therapeutic schemes ameliorated bradycardia and reduced the numbers of AVB2 events induced by T. cruzi infection. Severe bradycardia and AVB2 are ECG changes associated with aggravation of the cardiac form of CD (26). Hence, it is conceivable to propose that PTX, Bz, and Bz-plus-PTX therapies may reduce the progression of electrical abnormalities in T. cruzi-infected patients. PTX and Bz plus PTX reduced the prolonged QTc interval induced by T. cruzi infection. Moreover, Bz-plus-PTX therapy reversed the prolonged QTc interval observed pretherapy (120 dpi) and restored an ECG profile resembling that of NI controls. Importantly, a prolonged QTc interval is a poor prognosis for mortality in Chagas' heart disease (41). Therefore, our data suggested that combination therapy with Bz plus PTX might offer an alternative to improve the cardiac condition of CD patients and contribute to better prognosis.

Several reports point to the involvement of the immune response in the success of treatment with Bz in T. cruzi-infected mice (42, 43). In addition, a subcurative dose of Bz (25% of the curative dose) associated with Th1 immunostimulators (interleukin-12 [IL-12] or gamma interferon [IFN-γ]) increased survival and reduced cardiac lesions (36, 42, 43). However, our previously reported data support that the host immune response following T. cruzi infection is implicated not only in the control of parasite replication and beneficial effects on cardiac lesions but also in immunopathology (3, 9, 18, 22). Previously, PTX was demonstrated to ameliorate cardiac dysfunction in patients with noninfectious heart disease in association with downmodulation of inflammatory biomarkers such as plasma TNF concentrations (16, 17). PTX improved heart injury and electrical and functional heart abnormalities in mice with CCC without interfering with TNF levels but reducing TNFR1 expression (18). TNFR1 signaling mediates most of the biological activities of TNF (28, 29). Furthermore, TNF, by acting on TNFR1 but not TNFR2, aggravates noninfectious cardiopathy (29). Indeed, in a model of angiotensin II-induced heart pathology, TNFR1 contributes to heart fibrosis (44). Although TNFR1 participates in T. cruzi control during the acute phase (45), in chronic infection, the abrogation of TNFR1 expression in PTX-treated mice did not interfere with parasite control (18). Therefore, our data showing the simultaneous reduction of TNF expression in the heart tissue and TNFR1 expression on CD8^+^ T cells in Bz-plus-PTX-treated mice suggest that the combination therapy has an additional effect of the individual use of Bz or PTX as a modulator of TNF and TNFR1 expression. Furthermore, these data support that the TNF/TNFR1 signaling pathway is not crucial for parasite control during chronic infection but might promote heart tissue injury and electrical alterations, as previously proposed (9). In contrast to PTX therapy, which had no effect, Bz treatment decreased sTNFR1 levels in serum. The presence of sTNFR1 and sTNFR2 in extracellular fluids denotes shedding of transmembrane receptors (46). Notably, concentrations of sTNFRs in serum have been shown to reflect the severity of cardiac disease (47) and can be a powerful biomarker of heart tissue injury (48). Importantly, in chronically infected mice treated with Bz plus PTX, there is a complementary effect of PTX and Bz on the expression of TNF, TNFR1, and sTNFR1, which might contribute to a better prognosis for CCC in mice administered the combination therapy. Thus, in experimental chronic Chagas' heart disease, the effects of Bz were apparently supplemental to the previously shown beneficial effects of PTX therapy (18).

In a model of sepsis, Bz used at a subcurative dose (25 mg/kg) showed immunomodulatory properties by inhibiting the expression of NF-κB and mitogen-activated protein kinase (MAPK) (p38 and extracellular signal-regulated kinase [ERK]), which are crucial for TNF and iNOS/NOS2 expression and NO production (27). Indeed, our data show significant reductions in TNF mRNA expression levels in heart tissues of chronically T. cruzi-infected mice after treatment with Bz or Bz plus PTX. Importantly, TNF favors T. cruzi infection in nonprofessional epithelial cells (49). Moreover, IFN-γ-induced TNF and NO fuel astrocyte infection by T. cruzi (50). Thus, the Bz-triggered reduction in TNF levels may also interfere in this vicious TNF-fueled parasite growth circuit, contributing to T. cruzi elimination in chronic infection. Additionally, both Bz and PTX reduced TNFR1 expression levels. This outcome suggests that Bz and PTX may act on a common signaling pathway, such as NF-κB (27), or on different pathways with the same effect; however, the molecular mechanisms remain to be clarified.

TNF upregulates T. cruzi-triggered NO production by cardiomyocytes. Although NO is a trypanocidal agent in the acute phase (7), its reduction during the chronic infection, particularly the drastic decrease after Bz-plus-PTX therapy, did not hamper parasite control in chronically infected mice. These findings support the idea that NO modulation in the chronic phase of T. cruzi infection is a reasonable goal (12). Plasma NO levels have been associated with the severity of Chagas' heart disease (5, 6). Furthermore, NO produced via iNOS/NOS2 activity has been implicated in heart injury in chronically T. cruzi-infected monkeys and mice (12, 22). A recent study reported that a low dose of Bz (25 mg/kg/day) administered to acutely T. cruzi-infected mice resulted in parasite clearance and attenuated heart lesions with reduced expression levels of the inflammatory cytokines IL-1β and IL-6 and iNOS/NOS2 in heart tissue (51). Furthermore, *in vitro* treatment of T. cruzi-infected cardiomyocytes with Bz reduced NO_x_ production and expression of iNOS/NOS2, IL-1, and IL-6 associated with inhibition of the NF-κB pathway (51). The mechanisms underlying the improvement of heart conditions after Bz-plus-PTX therapy remain uncertain. It is possible that the beneficial effects of the combination therapy are due to an intervention in a circuit triggered by T. cruzi infection, leading to an increase in the expression level of TNF. In this vein, TNF may promote parasite infection and growth as well as augment the expression of TNFR1 and, consequently, TNF/TNFR1 signaling, fueling NO production, which may also involve the NF-κB pathway. Hence, Bz-plus-PTX combination therapy potentially inhibits the harmful immune circuits involved in parasite persistence and heart injury but preserves immunity against the parasite.

Based on our initial hypothesis and supported by the findings described above, we predicted that the beneficial action of Bz-plus-PTX administration to chronically T. cruzi-infected mice would persist after therapy interruption. Indeed, our data showed that the Bz-plus-PTX combination hampered the progression of electrical abnormalities such as atrial flutter and AVB2. Atrial fibrillation and AVB2 are associated with Chagas' heart disease progression (26). In a follow-up study of CD patients, the frequencies of patients with AVBs and flutter were significantly higher among nonsurvivors than among survivors (52). Finally, only Bz-plus-PTX therapy sustained the beneficial effects on ECG abnormalities by reversing the prolonged QRS and QTc intervals induced by T. cruzi infection. Increased QRS intervals were previously associated with the severity of fibrosis and reduction of the left ventricular ejection fraction in CD patients (53). Moreover, a prolonged QTc interval has been described as a risk factor for death in CD patients (41). Therefore, our data support that combination therapy with Bz plus PTX administered to mice in an experimental model of chronic T. cruzi infection interfered with crucial aspects of chronic Chagas' heart disease, improving prognosis.

A multicenter and randomized 5-year follow-up study and a systematic review of the literature with a meta-analysis indicate that the efficacy of treatment with Bz in late chronic T. cruzi infection to reduce clinical events is doubtful, although parasite control is achieved (19, 54). Although 31% of chronically infected patients in a clinical trial with the optimal dose of Bz abandoned treatment due to adverse drug reactions, the curative dose of Bz was considered safe (55). Here, we provide evidence that PTX emerges as an adjuvant, combined with a suboptimal dose of Bz (25% of the curative dose), to treat CCC. Besides the use of PTX to treat peripheral vascular disease, this drug has received new indications as an anti-inflammatory and cardioprotective agent as well as an adjuvant to treat diabetic nephropathy (16, 17, 56, 57). In CD patients, the use of PTX has not yet been explored. However, many years of clinical experience indicate that this drug has a favorable profile; therefore, if it is demonstrated to be efficacious, it could be an attractive medicament to treat patients with Chagas' heart disease. Nevertheless, Bz-plus-PTX combination therapy is a rational strategy based on interference in parasite load and detrimental immunological circuits to reduce the dose, treatment period, and toxicity of Bz, aiming to reduce the progression of chronic Chagas' heart disease. Furthermore, the sustained beneficial effect of Bz plus PTX on crucial aspects of CCC may predict a better prognosis for CD patients administered this combination therapy. Thus, our data support a clinical trial using Bz-plus-PTX combination therapy based on the paradigm that eradication of chronic infection is not a requirement to open an opportunity toward a better quality of life for CD patients.
